# Resistance Exercise After Laparoscopic Surgery Enhances Improvement in Exercise Tolerance in Geriatric Patients With Gastrointestinal Cancer

**DOI:** 10.7759/cureus.15454

**Published:** 2021-06-05

**Authors:** Kohei Tanaka, Ayano Taoda, Hirohiko Kashiwagi

**Affiliations:** 1 Rehabilitation Medicine, Osaka Police Hospital, Osaka, JPN

**Keywords:** gastrointestinal cancer surgery, skeletal muscle index, physical function, exercise capacity, cancer rehabilitation

## Abstract

Introduction: Early mobilization after cancer surgery is generally recommended. However, the effects of postoperative resistance exercise during a hospital stay have been rarely investigated. This study aimed to clarify the effects of resistance exercise after laparoscopic surgery on exercise tolerance and skeletal muscle mass in geriatric patients with gastrointestinal cancer.

Methods: This single-center retrospective observational study included patients with gastrointestinal cancer who admitted for laparoscopic surgery. Exercise tolerance and skeletal muscle mass were assessed using a six-minute walking test (6MWT) and skeletal muscle index (SMI) at admission and discharge, respectively. Intergroup comparisons of absolute changes in 6MWT and SMI were analyzed by the unpaired t-test or Mann-Whitney U test according to whether resistance exercise was performed or not. Multivariable linear regression analyses were used to analyze the association of resistance exercise with changes in 6MWT and SMI. The analyses were adjusted for age, sex, cancer stage, postoperative complication, and preoperative exercise tolerance and skeletal muscle mass.

Results: Altogether, 66 patients (mean age 69.9 years; 60.6% men) were recruited. Of them, 72.7% performed the resistance exercise and started at a median of 4.5 postoperative days. There were no significant intergroup differences in absolute changes in the 6MWT and the SMI (p = 0.153, p = 0.476, respectively). Multivariable linear regression analysis showed that resistance exercise was independently and positively associated with the 6MWT at discharge (*β *= 1.70, 95% confidence interval, 1.88 to 71.09) and did not significantly contribute to the SMI at discharge (95% confidence interval, -0.21 to 0.27).

Conclusion: Resistance exercise may enhance the improvement in postoperative exercise tolerance in geriatric patients with gastrointestinal cancer who underwent laparoscopic surgery.

## Introduction

Early postoperative mobilization is recommended as one of the critical criteria of the enhanced recovery after surgery (ERAS) protocol [1]. The ERAS protocol in patients with gastrointestinal cancer reduced postoperative complication rates and shortened length of hospital stay [2]. Skeletal muscle loss and deterioration of muscle strength and/or physical function occur frequently after surgery, thereby contributing to adverse outcomes such as higher incidence of postoperative complications, prolonged hospital stay, decreased quality of life and worse survival [3-5]. Some patients may start postoperative chemotherapy, and low skeletal muscle mass and physical weakness were reported as risk factors for chemotherapy toxicity [6-8]. Therefore, maintaining physical function and skeletal muscle mass should be the aim of postoperative physical therapy. However, since most studies on postoperative early mobilization focused on postoperative complications and length of hospital stay as outcomes, the effects of early mobilization on physical function are rarely investigated.

In a previous study, facilitating postoperative mobilization and increasing step count were not shown to be effective in the improvement of walking capacity [9]. This study suggests that simply walking in the confined environment of the hospital may not provide sufficient stimulation to induce a positive response in these patients. Schram et al. proposed a new alternative for postoperative physical activity intervention which is a supervised progressive resistance exercise program adapted to physical ability (either lying, sitting, or standing) and reported that it is feasible in the early postoperative period [10]. With few studies on the effects of exercise in the early postoperative period, it remains unclear whether resistance exercise is an effective strategy to enhance the improvement of postoperative physical activity in patients with gastrointestinal cancer. Laparoscopic surgery is less invasive and may enable patients to perform active exercises early in the postoperative period [10,11]. On the other hand, resistance exercise can be hindered by poor adherence caused by surgery-induced pain and fatigue in the clinical setting. Resistance exercise after surgery seems to have beneficial effects on physical function and skeletal muscle mass; however, the effects remain largely unknown.

Thus, this study aimed to clarify the effects of postoperative resistance exercise on exercise tolerance and skeletal muscle mass in patients who underwent laparoscopic surgery for gastrointestinal cancer.

## Materials and methods

Study population

This retrospective observational study was conducted at the Osaka Police Hospital. Medical records were retrospectively reviewed, and patients with gastric or colorectal cancer who were hospitalized for laparoscopic surgery from April 2019 to March 2020 were enrolled. Patients who had a history of cerebrovascular disease were excluded. This study was approved by the ethics committee of the Osaka Police Hospital (approval number 1021). We used the website of the hospital as an opt-out method to provide opportunities for participants to decline participation in this study.

Assessments

Physical function and body composition were assessed on the day before surgery and the day before discharge. The main outcomes were the six-minute walking test (6MWT) as an indicator of exercise tolerance and skeletal muscle index (SMI) as an indicator of skeletal muscle mass. The 6MWT was measured using a 30-m straight course. The SMI and other indicators of body compositions (body mass index, skeletal muscle mass, body fat mass, and body fat percentage) were measured using InBody 770 (InBody Japan, Japan). Other indicators of physical function were handgrip strength, gait speed, and a five-time sit-to-stand test. Handgrip strength was measured twice on each side, and the maximum value was recorded. Comfortable gait speed was calculated from the time required to walk 4 m. Maximum gait speed was measured at 10 m. The cancer stage was defined according to the Union for International Cancer Control TNM classification. Postoperative complications until discharge were classified according to the Clavien-Dindo classification.

Physical therapy

 Postoperative physical therapy was started on the first postoperative day, wherein the first step was assisting the patient to get out of bed and extending the walking distance. Subsequently, the physical therapist facilitated the patient to start resistance exercise early as possible after the surgery and adjusted the stratification of modalities (from bed to sitting to standing). The physical therapist recommended that the patient prioritize bodyweight resistance exercises such as squats, lunges, and calf raises in the standing position instead of exercises in the supine or sitting position. The exercise load was set to be light to moderate in subjective exercise intensity as determined by the Borg Scale (11 to 13 on a scale of 20). Physical therapists facilitated patients to perform at least one set of 10 repetitions of each exercise and encouraged progression by adding more sets when patients were found to be easily completing 10 repetitions. The first day of resistance exercise was defined as the day on which at least one set of bodyweight resistance exercise was performed in the standing position.

Statistical analysis

Data are presented as mean ± standard deviation. All data were analyzed using the SPSS software (IBM SPSS Statistics for Windows, Version 21.0, IBM Corporation, NY, USA). Patients were divided into two groups according to whether resistance exercise was performed or not after surgery. Based on the results of the Shapiro-Wilk test, intergroup comparisons were analyzed by unpaired t-test or Mann-Whitney U test for the baseline characteristics and absolute changes in physical function and body composition. A chi-squared test was used for testing the goodness of fit of the baseline characteristics. Multivariable linear regression analysis was used to analyze the association of resistance exercise with the 6MWT and SMI at hospital discharge. The analyses were adjusted for age, sex, cancer stage, postoperative complication, and preoperative 6MWT and SMI. Multicollinearity was assessed using the variance inflation factor. A variance inflation factor value from 1 to 10 was considered the absence of multicollinearity. Missing data were excluded from the analysis. The statistical significance was set at p < 0.05.

## Results

We recruited 66 patients in this study. The baseline characteristics of the study population are shown in Table 1. Postoperative physical therapy was started on the first postoperative day, and 84.8% (56/66) of the patients were able to take their first walk on that day. The other 10 patients could perform their first walk within a few postoperative days. Resistance exercises were started at a median of 4.5 (interquartile range: 3.0-7.0) days, and 72.7% (48/66) of the overall patients were able to perform them during their hospital stay.

**Table 1:** Baseline characteristics and intergroup comparisons according to the type of exercise performed.

Characteristics	Total participants (n = 66)	Resistance exercise		P-value
		With (n = 48)	Without (n = 18)	
Age (years)	69.9 ± 10.2	69.9 ± 9.6	69.7 ± 11.5	0.902
Sex, n (%)				
Male	40 (60.6)	34 (70.8)	6 (33.3)	0.010
Female	26 (39.4)	14 (29.2)	12 (66.7)	
Cancer stage, n (%)				
1	24 (36.4)	18 (37.5)	6 (33.3)	0.850
2	20 (30.3)	15 (31.3)	5 (27.8)	
3	17 (25.8)	11 (22.9)	6 (33.3)	
4	5 (7.6)	4 (8.3)	1 (5.6)	
Clavien-Dindo classification, n (%)				
0	55 (83.3)	41 (85.4)	14 (77.8)	0.835
1, 2, 3a	10 (15.2)	6 (12.5)	4 (22.2)	
3b	1 (1.5)	1 (2.1)	0 (0)	
Length of postoperative stay (days)	12.2 ± 6.7	12.6 ± 7.0	11.3 ± 6.0	0.127
Body mass index (kg/m^2^)	22.8 ± 3.3	23.2 ± 3.5	21.8 ± 2.6	0.117
Skeletal muscle mass (kg)	24.1 ± 6.3	25.2 ± 6.6	21.0 ± 4.0	0.014
Skeletal muscle index (kg/m^2^)	6.8 ± 1.2	7.0 ± 1.2	6.3 ± 1.0	0.031
Body fat mass (kg)	15.6 ± 6.0	16.3 ± 6.5	13.9 ± 4.0	0.205
Body fat percentage (%)	25.7 ± 6.5	25.5 ± 6.6	26.1 ± 6.1	0.756
Albumin (g/dL)	4.1 ± 0.4	4.1 ± 0.4	4.1 ± 0.3	0.751
Total lymphocyte count (K/µL)	1.8 ± 0.6	1.8 ± 0.1	1.7 ± 0.1	0.741
Total cholesterol (mg/dL)	194.6 ± 36.8	191.9 ± 36.8	201.9 ± 35.6	0.334
Hand grip strength (kg)	28.7 ± 9.2	30.0 ± 9.3	25.2 ± 8.0	0.061
Comfortable gait speed (m/s)	1.03 ± 0.22	1.05 ± 0.22	1.00 ± 0.21	0.398
Maximum gait speed (m/s)	1.59 ± 0.40	1.59 ± 0.41	1.59 ± 0.38	0.940
Five-time sit-to-stand test (s)	9.4 ± 2.4	9.4 ± 2.4	9.4 ± 2.5	0.965
Six-minute walking test (m)	437.5 ± 97.0	445.0 ± 95.8	415.0 ± 96.6	0.265

Table 2 shows the results of the absolute changes in each variable from admission to discharge. Most variables decreased postoperatively. The decrease in maximum gait speed in participants who performed resistance exercises was significantly lower than in those who did not (p = 0.016). There were no significant differences in the absolute changes in other variables between the patients with or without resistance exercise.

**Table 2:** The comparisons of the changes in body composition and physical function between groups according to the type of exercise performed.

Absolute changes	Resistance exercise		P-value	
	With (n = 48)	Without (n = 18)		
Skeletal muscle mass (kg)	-1.3 ± 1.2	-0.9 ± 0.8		0.302
Skeletal muscle index (kg/m^2^)	-0.6 ± 0.4	-0.5 ± 0.3		0.476
Body fat mass (kg)	-1.7 ± 1.4	-1.4 ± 1.2		0.403
Body fat percentage (%)	-1.0 ± 2.5	-1.2 ± 2.0		0.880
Handgrip strength (kg)	-1.1 ± 2.3	-1.3 ± 2.4		0.324
Comfortable gait speed (m/s)	0.01 ± 0.18	-0.05 ± 0.23		0.310
Maximum gait speed (m/s)	-0.12 ± 0.25	-0.29 ± 0.39		0.035
Five-time sit-to-stand test (s)	0.1 ± 2.0	0.6 ± 2.4		0.459
Six-minute walking test (m)	-31.6 ± 49.8	-54.2 ± 68.7		0.153

The results of the multivariable linear regression analysis of the 6MWT and SMI are shown in Tables 3 and 4, respectively. The analysis showed that the resistance exercises were independently and positively associated with the 6MWT at hospital discharge (β = 1.70; 95% confidence interval, 1.88 to 71.09). Resistance exercise was not significantly associated with the SMI at hospital discharge (95% confidence interval, -0.21 to 0.27).

**Table 3:** Multivariable linear regression analysis on the six-minute walking test at hospital discharge. *R*^2^ = 0.686, Adjusted *R*^2^ = 0.647, p < 0.001. SMI: skeletal muscle index, 6MWT: six-minute walking test.

Variables	Coefficients		p-value	95% Confidence interval			Variance inflation factor
	B	β					
Sex	-6.33	-0.32	0.788	-53.19,	40.52	2.56	
Age	0.43	0.05	0.594	-1.18,	2.04	1.32	
Cancer grade	-4.76	-0.05	0.542	-20.29,	10.77	1.10	
Clavien-Dindo classification	-15.10	-0.13	0.101	-33.26,	3.05	1.11	
Pre SMI	-6.76	-0.08	0.489	-26.19,	12.68	2.49	
Pre 6MWT	0.77	0.78	< 0.001	0.59,	0.95	1.44	
Resistance exercise	36.49	1.70	0.039	1.88,	71.09	1.18	

**Table 4:** Multivariable linear regression analysis of the skeletal muscle index at hospital discharge. *R*^2^ = 0.903, Adjusted *R*^2^ = 0.889, p < 0.001. SMI: skeletal muscle index, 6MWT: six-minute walking test.

Variables	Coefficients		P-value	95% Confidence interval			Variance inflation factor
	B	β					
Sex	-0.21	-0.10	0.188	-0.53,	0.11	2.67	
Age	-0.01	-0.04	0.427	-0.02,	0.01	1.15	
Cancer grade	-0.06	-0.05	0.282	-0.17,	0.05	1.11	
Clavien-Dindo classification	-0.10	-0.08	0.098	-0.21,	0.02	1.13	
Pre SMI	0.77	0.82	< 0.001	0.63,	0.91	2.65	
Pre 6MWT	0.01	0.08	0.156	0.00,	0.00	1.34	
Resistance exercise	0.03	0.01	0.782	-0.21,	0.27	1.12	

## Discussion

The major finding from the present study is that resistance exercise has a significantly positive effect on the 6MWT at discharge in postoperative geriatric patients with gastrointestinal cancer but not on the SMI at discharge.

The decline in muscle strength and physical function is commonly observed after cancer surgery [12,13]. Postoperative physical weakness may be affected by several factors including the inflammatory response to the surgical invasion, and deterioration of cardiopulmonary and neuromuscular functions. Certainly, the inflammatory response is reported to be associated with impaired physical function [14,15]. Previous studies also reported that functional residual capacity is decreased and neuromuscular function is impaired after surgery [16,17]. The 6MWT is a practical test, which is valid and is commonly used to measure exercise tolerance in the clinical setting [18]. The result of multivariable linear regression analysis showed resistance exercise was significantly and positively related to 6MWT at hospital discharge. It is reported that increasing step count is not effective in the improvement of walking capacity [9]. Although it may be difficult to cause morphological changes such as muscle hypertrophy with low-intensity resistance exercise, repeated muscle contractions might stimulate neuromuscular activation and improve physical function. The activation of the neuromuscular system is associated with increased motor units and increased firing frequency. Previous studies reported that resistance exercise, even at low intensity, improves exercise tolerance [19]. The improvement in physical function with resistance training in the early phase is considered to be a result of neural adaptations and may be associated with an increase in motor units [20,21]. Resistance exercise was shown to be significantly and positively related to 6MWT at hospital discharge. This result suggests that even low-intensity resistance exercise with bodyweight may be effective in enhancing the improvement of walking capacity after surgery. Facilitating patients to start bodyweight resistance exercises by physical therapists in the early postoperative period may be a clinically important intervention for patients with gastrointestinal cancer.

The skeletal muscle is characterized by high plasticity and responds to various stressors including surgical invasion, malnutrition, metabolic abnormalities, and physical inactivity. Skeletal muscle loss is a critical concern as it may lead to adverse clinical outcomes [4,5]. However, preventing skeletal muscle loss after surgery seems to be difficult. Surgical invasion is a critical factor for enhancing skeletal muscle catabolism [22]. Surgical invasion induces an inflammatory response, which increases inflammatory cytokines and reprioritization of the hepatic protein synthesis, and the inflammatory response increases skeletal muscle catabolism and suppresses skeletal muscle anabolism [23-25]. Surgical invasion may induce postoperative insulin resistance, and insulin resistance contributes to protein catabolism due to glycogenesis. A previous study showed that postoperative protein supplementation is insufficient to attenuate protein catabolism [26]. In the clinical setting, reduced food intake and digestive dysfunction are commonly observed in patients with gastrointestinal cancer after surgery, and nutritional intake is frequently inadequate. It is reported that the decrease in energy intake, especially insufficient protein intake, induces skeletal muscle loss [27]. Although resistance training is a well-known method of stimulating the skeletal muscle anabolic reaction [28], it has been reported that skeletal muscle in older adults is less responsive to exercise than in younger adults [29]. A recent study reported that exercise programs for older adults have a significant positive effect on physical performance, but not on skeletal muscle mass [30]. In the present study, postoperative resistance exercise did not show the preventative effect on skeletal muscle loss. The short-term effects of postoperative resistance exercise for patients with gastrointestinal cancer remain largely unknown. Although it may be difficult to suppress surgery-induced acute skeletal muscle loss during the early postoperative period, further studies are needed to clarify the effects of postoperative resistance exercise.

Several limitations were existing in this study. First, this study did not account for the intensity, repetition, or frequency of resistance exercise in the statistical analysis. Some patients may have exercised voluntarily in addition to physical therapy. The effects of exercise may be greatly dependent on the dose of exercise. Future research is needed to examine the dose-dependent effects of resistance exercise on physical function and skeletal muscle mass. Second, this is a retrospective observational study, and it was not decided randomly in advance whether resistance exercises would be performed or not. In the future, prospective studies with standardized exercise programs are necessary to clearly verify the effects of exercise.

## Conclusions

Postoperative bodyweight resistance exercise enhanced improvement in exercise tolerance after laparoscopic surgery in patients with gastrointestinal cancer but did not suppress skeletal muscle loss. Facilitating patients to start resistance exercise early in the postoperative period by physical therapists may be a clinically effective strategy to improve exercise tolerance. Continuous exercise after hospital discharge would be required to increase skeletal muscle mass in particular.
